# Supplementary material for: Effects of the Blending Ratio on the Design of Keratin/Poly(butylene succinate) Nanofibers for Drug Delivery Applications
Source: Biomolecules. 2021 Aug 12;11(8):1194. doi: 10.3390/biom11081194 (PMC8392087; doi:10.3390/biom11081194)
Supplement: Supplementary file 1 [file biomolecules-11-01194-s001.zip › biomolecules-1264192-supplementary.pdf]

Supplementary Materials

Type of the Paper

Effects of the blending ratio on the design of keratin/poly(buthylene succinate) nanofibers for drug delivery applications.

Giulia Guidotti[1§], Michelina Soccio[1§], Edoardo Bondi[1], Tamara Posati[2], Giovanna Sotgiu[2,4], Roberto Zamboni[2], Armida Torreggiani[2], Franco Corticelli[3], Nadia Lotti[1,*] and Annalisa Aluigi[2,4*].

[1] Department of Civil, Chemical, Environmental, and Materials Engineering, University of Bologna, Via Terracini, 28, 40131, Bologna, Italy;
[2] Institute of Organic Synthesis and Photoreactivity – Italian National Research Council, Via P. Gobetti, 101, 40129, Bologna, Italy;
[3] Institute for Microelectronics and Microsystems – National Research Council, via P. Gobetti 101 40129 Bologna, Italy
[4] Kerline srl
* Correspondence: Annalisa Aluigi: annalisa.aluigi@isof.cnr.it; Nadia Lotti: nadia.lotti@unibo.it;
§ These Authors contributed equally

S.1 Shear viscosity of the solutions of keratin in *1,1,1,3,3,3-Hexafluoro-isopropanol* (HFIP)

In the our previous work, keratin solutions in HFIP at 15%wt were used for the preparation of keratin/PBS blend solution 50/50[1]. However, this concentration is at the limit of the keratin solubility in HFIP, since at slightly higher concentration the protein precipitation occurs. Therefore, in this work the keratin concentration used to prepare the electrospinning solution was slightly reduced to 13%. The rheological behaviour of keratin/HFIP solution at 13% wt of keratin is very close to that of the 15%wt solution as shown in figure S1.

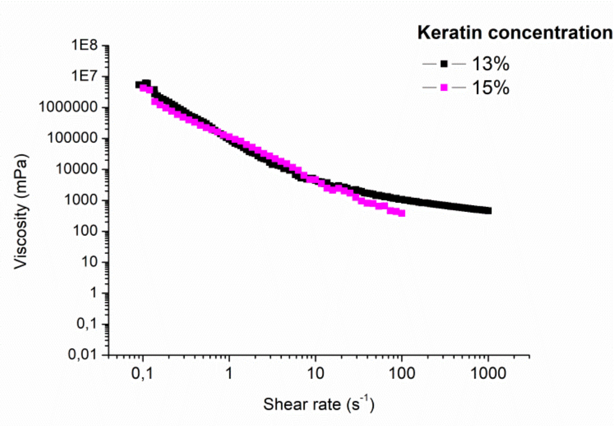

Figure S1. Shear viscosity of keratin solutions in HFIP at 13%wt and 15% wt

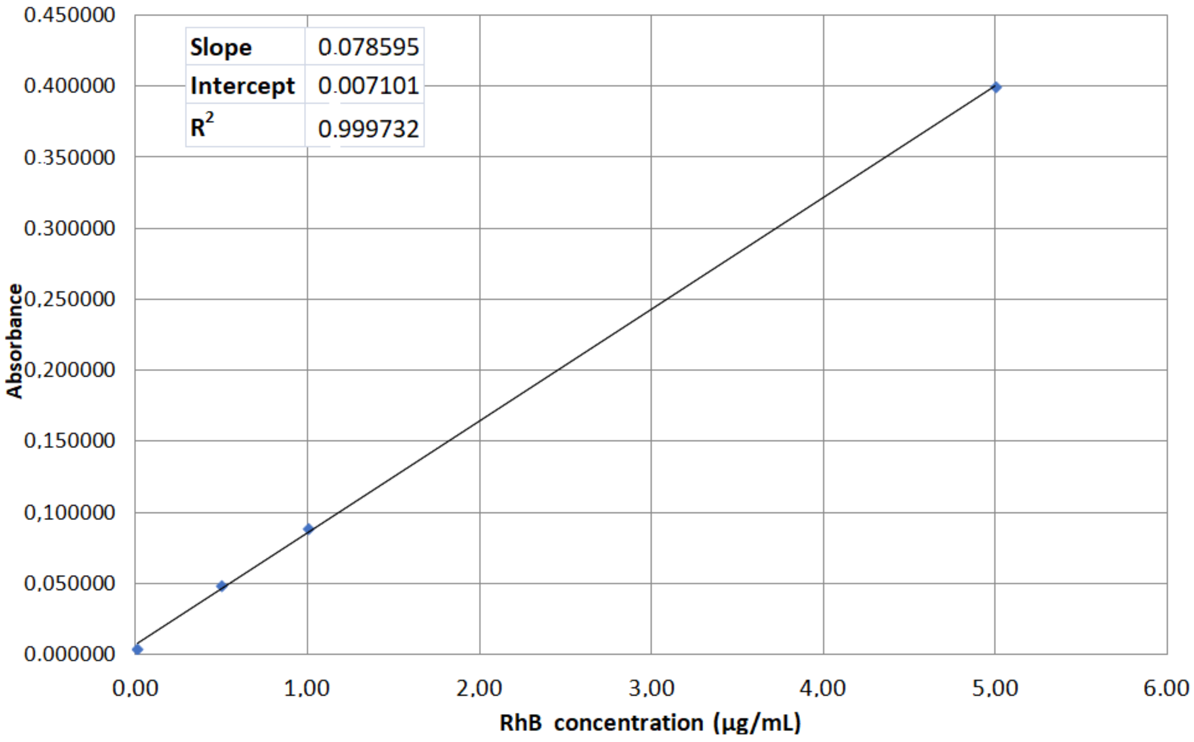

Figure S2. Calibration curve of Rhodamine B in phosphate buffer pH 7.4

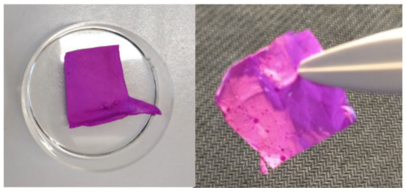

Figure S3. Free standing nanofibrous mats of keratin/PBS 50/50

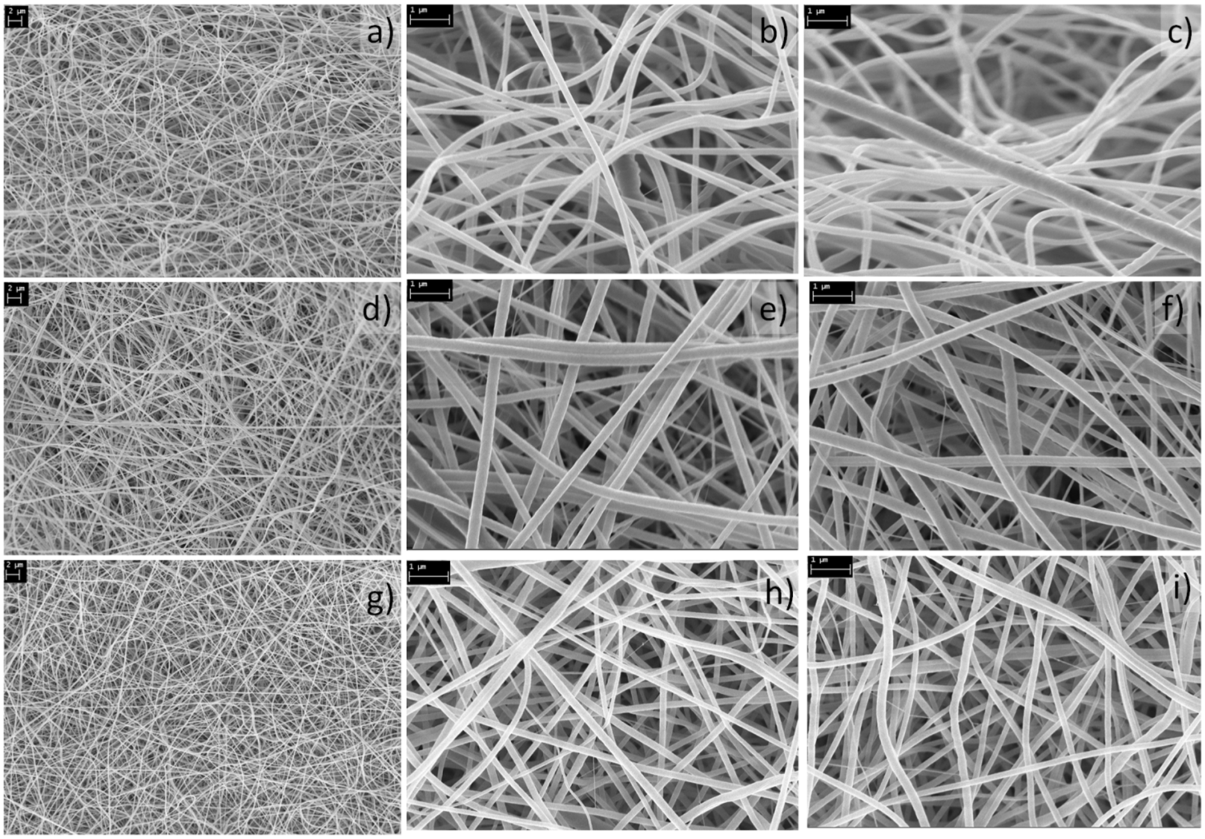

Figure S4. Keratin/PBS blend nanofibers loaded with RhB (3% wt) at different magnification: 30/70 (a,b,c); 50/50 (d,e,f) and 70/30 (g,h,i)

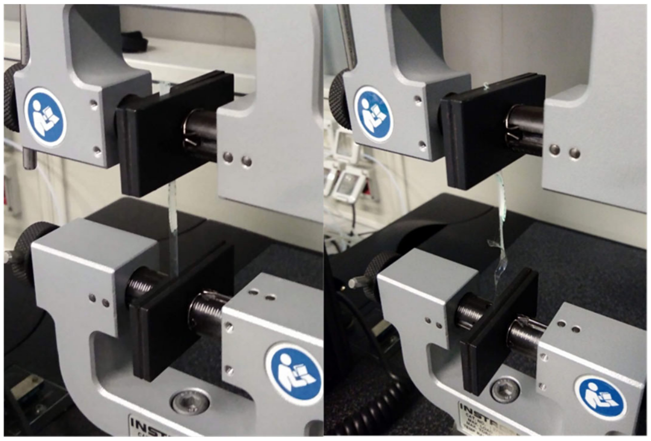

Figure S5. *Pictures of Keratin/PBS50/50 (left) and PBS (right) electrospun mats during shear strength measurements.*
